# Bilingualism as a Slice of Swiss Cheese

**DOI:** 10.3389/fpsyg.2021.769323

**Published:** 2021-11-08

**Authors:** Ellen Bialystok

**Affiliations:** Department of Psychology, York University, Toronto, ON, Canada

**Keywords:** bilingualism, cognition, lifespan, reductionism, individual differences

## Introduction

As the Covid-19 pandemic ravaged the global population, an intense discussion began about how best to contain the spread of the deadly virus. Debates ensued about whether masks should be mandated, borders closed, crowds controlled, businesses shuttered, and so on. The assumption in these debates was that there was a correct solution that would solve the problem and allow life to return to normal, the only issue being to determine which of the proposed mitigations would best achieve that goal. The discussions quickly became political, with dogmatic positions being asserted on all sides.

Although many public health officials advocated for enforcing all such interventions, there was nonetheless an underlying sense of priorities, such as focusing on mask mandates so other approaches, such as closing schools or businesses, could be avoided. There was little evidence to support these assumptions and no logic provided for why they were chosen. But in the midst of these high-stakes discussions, a virologist, Ian Mackay, took a different view (described in Lewis, 2021). Following earlier work by Reason (1990), he argued that all interventions have imperfections and the most effective means of avoiding the imperfections in each is to combine them so the weakness in one approach is compensated by a strength in another. Reason compared this approach to a package of Swiss cheese: each slice has holes, but the holes are in different places, so when the slices are stacked together, all the holes are blocked. The problem Reason addressed was how to manage inevitable human error to avoid devastating accidents. His solution was that each attempt blocks a different hazard, so ultimately, it is in the combination, or as he called it “system,” that safety is achieved (Reason, 2000). Mackay's contribution was to apply this approach to the mitigation of disease in a pandemic: no single solution alone will halt the spread of the disease but all approaches in combination will be effective.

And so it is with bilingualism. For about a decade there has been fierce debate about whether bilingualism improves cognitive systems and brain structures. The debate is polarized, aggressive, and unresolved. On one side, researchers argue that empirical evidence from multiple sources has demonstrated that bilingual participants outperform monolinguals on a range of cognitive tasks, with most discussion focused on executive functions (Baum and Titone, 2014; Bialystok, 2017; Antoniou, 2019); those on the opposite side argue that attempts to replicate those experiments fail to reveal group differences so the reported differences must be spurious (Paap and Greenberg, 2013; von Bastian et al., 2016). Moreover, meta-analyses of the same body of research have supported both the validity of the positive claims (van den Noort et al., 2019; Grundy, 2020) and null conclusions in which there is no relation between bilingualism and cognitive level (Lehtonen et al., 2018; Donnelly et al., 2019). Although similar issues apply to the possible role of bilingualism in modifying brain structure and brain networks, those debates are less passionate and the evidence less controversial, so the present discussion will focus on the behavioral evidence connecting bilingual experience to behavioral outcomes. How can there be so much uncertainty about the relation between an identifiable experience and a set of measurable cognitive outcomes? Can the Swiss cheese model help us to understand this debate?

## Relation Between Bilingualism and Cognition

The present argument is that the debate rests on a reductionist error in which both the definition of bilingualism and the nature of cognitive ability it allegedly modifies are oversimplified, thereby reducing the relation between them to a single-factor description. The central concept, bilingualism, is treated as a binary notion by opposing it to another oversimplification, monolingualism. Moreover, the evidence relating this binary notion to a set of outcomes is objectified and assigned a name, “The Bilingual Advantage.” Once something has been concretized in this way it can be treated as an entity that exists or does not exist; all nuance evaporates. The test for reductionism is to replace a concept with its definition by inserting the phrase “nothing but.” In this way, bilingualism is *nothing but* the ability to speak two languages and the cognitive consequence of bilingualism is *nothing but* superior performance on some executive function task. These concepts then take the form of a checklist: How many languages do you speak? What were the scores on the executive function tasks for these binary groups? Thus, when the group designated as “bilingual” fails to excel in some cognitive task designated as “executive function,” the conclusion is that there is no relation between the concepts (see for example Nichols et al., 2020). But life rarely presents such discrete options.

Why should bilingualism have any relation to cognitive outcomes? There is no obvious reason to assume that a linguistic experience, bilingualism, would impact non-verbal cognitive outcomes. Indeed, most research investigating transfer of skills across domains shows weak evidence for this possibility, indicating at best only near transfer across similar abilities (Shipstead et al., 2012; Simons et al., 2016). However, it is well-established that both languages are simultaneously active in bilingual minds, even in strongly monolingual contexts, creating ongoing potential conflict (Kroll et al., 2012). Since bilinguals rarely make intrusion errors (Gollan and Ferreira, 2009), some mechanism must be responsible for managing attention to the target language while excluding the unwanted language. The general view is that this mechanism is based in the domain-general attention system used for executive functioning (Bialystok et al., 2009). Supporting evidence from imaging studies has demonstrated that overlapping attention networks are used both for language selection and non-verbal cognitive control (Luk et al., 2012; Wong et al., 2016). This use of general attention systems for language processing by bilinguals links the two domains and opens the possibility for interactions between them.

The argument, therefore, is that experience in managing two languages recruits the general attention mechanisms used for other cognitive activities, thereby changing them. However, demonstrating this relation empirically is complex largely because of the difficulty in defining “bilingualism.” In most psychological research in which groups are compared, the designation of the groups is objectively transparent and the tested outcome has a simple relation to group membership. Thus, studies can compare 4-year-olds and 6-year-olds on a cognitive reasoning task, younger and older adults on a memory recall task, men and women on a spatial processing task (although this binary is becoming increasingly complex), or musicians and non-musicians on an auditory perception task. Because the criteria for group membership are clear and the outcome task is related to a hypothesis about the difference between those groups, the results can be easily interpreted: older children are more cognitively advanced than younger children so outperform them on a reasoning task (Richland et al., 2006), younger adults have better memory than older adults so recall more items on specific memory tasks (Thomas and Hasher, 2012), men outperform women in spatial processing (Parsons et al., 2004), and musicians have more acute auditory perception than non-musicians (Boh et al., 2011). There is also a clear specificity in these relations: musical experience improves auditory acuity. In all these cases, too, studies sometimes show no difference between groups, and crucially, sometimes the expected effect is reversed, demonstrating younger children outperforming older children on a cognitive task (Otgaar et al., 2016) or older adults outperforming younger adults on a memory task (Castel, 2005). These exceptions are not taken as counterevidence to the general principle but rather as circumstances that reveal the inherent complexity in these behaviors without compromising the accepted difference between the groups.

Unlike other individual differences, bilingualism is not a binary category—it is a slice of Swiss cheese buried within a package of slices that together impact cognitive function. Sometimes the holes in the bilingualism slice are blocked by other slices that compensate for those gaps (high SES? Education?) but sometimes it is bilingual experience that is primarily responsible for the outcome presumably because of holes in the other slices (delay of symptoms of dementia?). At the risk of entering an infinite regress, bilingualism itself can be considered as a package of Swiss cheese, with different manifestations of bilingual experience placing the holes in different places that together define the experience. There is also ambiguity about the overall goal: Is it general cognitive ability, performance on specific cognitive tasks, or executive function ability? Finally, the mechanism for the relation in terms of attention across domains is less specific than the connection between musical experience and auditory acuity. Therefore, the complexity in understanding the relation between bilingualism and cognitive outcomes comes from defining bilingualism, defining the outcome, and identifying the mechanism that relates them. If the outcome of interest is the somewhat amorphous issue of developing and maintaining cognitive function across the lifespan, then the question is whether adding a slice of bilingualism has an impact on this cognitive package.

Explaining the factors that contribute to high functioning cognitive systems is surely at least as complex as defeating a viral pandemic. A single layer of cheese, such as the mask mandate, was never going to conquer the pandemic, and a single approach to boosting cognitive function, such as bilingual experience, cannot guarantee outcomes. There has never been a claim that this is a single-factor model in which bilingual experience is irrevocably responsible for better cognitive outcomes, but there is clear evidence that it *contributes* to those outcomes. Crucially, including that bilingualism slice is almost never associated with poorer cognitive outcomes. The implications of this metaphor are that bilingualism alone will not guarantee positive effects on cognition, but that overall outcomes are better when bilingualism is included. This summary fits well with the actual body of evidence.

## Where the Holes are

The central idea in the cheese metaphor is that each intervention will carry its own weaknesses—it will have holes. Anticipating where those holes are for bilingualism is especially challenging because each experience of bilingualism is different. Although such differences as age of acquisition of the additional language(s), duration of active bilingualism, intensity of use, proficiency in each language, and the like (Luk and Bialystok, 2013), have been acknowledged for some time, detailed examination of them has only recently become an important area of research. Thus, different cognitive outcomes have been reported for individuals who became bilingual early or later in life (Luk et al., 2011; Pelham and Abrams, 2014; Vega-Mendoza et al., 2015), were tested as children or adult bilinguals (Bialystok et al., 2005; Dash et al., 2019), and engaged in frequent language switching or not (Festman et al., 2010; Prior and Gollan, 2011, 2013; Verreyt et al., 2016). All these studies found a connection between specific aspects of bilingual experience and cognitive outcome, but the role of these features in modulating the results makes it difficult to propose general assertions about the relation between bilingualism *per se* and cognitive outcomes or the possible underlying mechanism responsible for those effects.

One approach to addressing variations in bilingualism is to quantify the experience in terms of some of these factors. In these studies, bilinguals are not necessarily compared to monolinguals (although they can be) but rather are positioned along a continuum of bilingualism. The gradient can be composed of a single factor, such as age of acquisition of the new language, or a range of factors including aspects of experience, language proficiency, and language use, as in the Language and Social Background Questionnaire (Anderson et al., 2018). The instrument elicits details about background, experience, use patterns and so on, to produce scores on three factors—home language use, social language use, second-language proficiency—which are then weighted to create an overall bilingualism score. Other instruments have been created for this purpose and achieve similar results (Marian et al., 2007; Li et al., 2014). In these studies, more experience with bilingualism is associated with both better test performance (Guerrero et al., 2016; Pot et al., 2018; DeLuca et al., 2020; Bialystok and Shorbagi, 2021) and better brain structure (Hervais-Adelman et al., 2018; Del Maschio et al., 2019; DeLuca et al., 2019; Sulpizio et al., 2020). These detailed associations undermine conclusions from binary procedures that classify participants in terms of their response to a simple question about how many languages they speak (Dick et al., 2019; Nichols et al., 2020) and have refined our understanding of the relation between bilingualism and cognition.

Recent studies have also described the role of the linguistic and sociolinguistic context in shaping bilingual experience and its effect on cognitive and brain outcomes. The most detailed model of this type is the Adaptive Control Hypothesis (Green and Abutalebi, 2013). The authors identify three interactional contexts in which two languages can be used and argue that each context engages different cognitive processes leading to different consequences for mind and brain. In a single language context, each language is used in a specific setting, as in one language at home and a different language at work. This context imposes few cognitive demands because there are multiple cues for language selection, so the main demand is to stay focused on the goal and select the correct language without interference from the other. The second context, dual language, is more challenging because both languages are used in the same setting but with different individuals. In addition to monitoring the language needed for this interlocutor, the context also requires switching, disengagement, and response inhibition to maintain focus on the correct language. Finally, dense code switching defines situations in which both languages are used by all individuals, so focus on the target language is less important. Because everyone can speak both languages, communication is not necessarily disrupted if there is a language switch. All three contexts require proficient bilingualism, but each places different demands on the cognitive systems needed to manage language use and so is associated with different outcomes.

Another approach to describing relevant differences in bilingual environments was proposed by Gullifer and colleagues through the notion of language “entropy” to reflect the variety and complexity of social situations in which both languages are used (Gullifer and Titone, 2020). They argue that greater social diversity of language use leads to a larger impact on the cognitive outcomes associated with bilingualism. By combining estimates of entropy with other individual differences, such as age of bilingual acquisition and intensity of bilingual experience, they offer a more complete account of the complexity of bilingualism (Gullifer et al., 2020). These metrics relate to brain structure in terms of functional connectivity while performing an executive function task (Gullifer et al., 2018) and overall better performance on that task (Gullifer and Titone, 2021). In short, the small variations in bilingual experience reflected in language entropy were positively associated with cognitive and brain outcomes.

These examples in which bilingual experience has been quantified in terms of the details of individual experiences and the situations in which the languages are used demonstrate the inadequacy of a monolithic concept called “bilingualism,” particularly one that is defined by its distinction from another monolithic concept, “monolingualism.” Even monolingualism exists in a context. Monolinguals living in a strongly homogeneous context, monolinguals living in a diverse context where bilingual use is prevalent, and bilinguals were taught a new language, Finnish; although word learning was similar across groups, electrophysiological responses to a phonetic feature distinctive in Finnish was only similar for bilinguals and monolinguals living in a diverse context (Bice and Kroll, 2019).

The argument to this point is that the complexity and diversity of bilingual experience rules out one-to-one mappings between bilingualism and cognitive performance. Instead, small differences in bilingual experience modify its relation to overall cognitive functioning. In this sense, bilingualism is a flawed intervention that nonetheless contributes to a larger goal.

## Reinforcing the Cognitive Dam

In the case of the multiple slices of Swiss cheese needed to mitigate a global pandemic, success was determined by some metric of pandemic severity. This outcome could be measured in several ways, such as number of cases, number of hospitalizations, or test positivity rate, and each outcome might be differentially impacted by each mitigation slice. And although the various outcome measures are likely correlated, they are not identical; number of cases is related to the number of hospitalizations but there are important differences between them. This multifaceted relation between individual mitigation strategies and the overall goal rules out simple interpretations, such as the effect of mask mandates on ending a pandemic, even though each slice contributes to that goal.

For bilingualism, there is a lack of clarity about the cognitive outcomes it is expected to impact. Although most of the literature has focused on executive function tasks, some studies have extrapolated these ideas to a range of cognitive abilities for which no relation to bilingualism would be expected. In general, there is no impact of bilingualism on verbal tasks or verbal conditions of cognitive tasks (Luo et al., 2013), simple tasks that can be performed with little effortful control (Comishen and Bialystok, 2021), and cognitive domains for which conflict resolution is not central, such as reasoning in the Tower of London Task (Papageorgiou et al., 2019). Yet, the absence of an effect in these cases is sometimes used to reject claims connecting bilingualism to cognition. In addition to explaining how each contributor to cognition works individually and defining its features, in this case, bilingualism, it is equally essential to set clear definitions for the outcome, in this case, cognition.

## Does Bilingualism Affect Cognitive Outcomes?

The implication of this perspective is that there is a real effect of bilingualism on cognitive function with a small effect size that can be overshadowed by other factors. But that is the way complex phenomena are determined. Drawing on research from genetics in which it eventually became clear that there was no simple mapping from single genes to outcomes, Gotz et al. (2021) argue that the same principles apply to psychological phenomena, including cognitive ability. They claim that complex psychological phenomena are determined by many factors, each of which typically has a small effect size, and that the search for a one-to-one relation between predictors and outcomes is reductionist and ultimately, incorrect. For this reason, attempts to isolate a single factor or interpret a complex outcome in terms of a single factor are misguided. Moreover, they argue that contrary to the usual assumptions, large effect sizes are likely more unreliable and unreplicable than are the small effects that may or not be statistically significant in a given study but are pervasive *across* studies. They implore researchers in psychology to reconsider the focus on large effect sizes and instead “reward accurate and meaningful effects rather than exaggerated and unreliable effects” (p. 5).

The effect of bilingualism on cognition is clearly in the range of small effect sizes. Most meta-analyses of this literature show an overall advantage for positive studies with a small effect size of about 0.15–0.20 before such corrections as publication bias or outlier removal are applied (see Grundy, 2020), but the result is interpreted in different ways: Some authors accept the significant effect and others argue that the effect size is not large enough to conclude that the positive results are reliable. However, these effect sizes are within the range found in meta-analyses of other moderating effects on cognition. For example, the effect size for the role of exercise on cognitive outcomes is between 0.10 and 0.25 (Etnier et al., 1997; Chang et al., 2012), yet there is no debate over the idea that exercise impacts cognitive outcomes. In that case, exercise might be another slice of Swiss cheese in the cognitive package, but the importance of its inclusion in the package is viewed positively.

The implication of this metaphor for understanding the effect of bilingualism on cognitive outcomes is that one cannot expect a simple relationship between the concepts. Bilingual experience has many varieties, and it is only one slice in a package that includes such factors as socioeconomic status, immigration status, cultural background, genetic endowment, general health, and such that impact cognitive outcomes. But the effect of bilingualism is real, and it contributes to the robustness of cognitive ability. And just as it is important to understand the way factors such as socioeconomic status impact cognitive level, so too it is essential to understand how bilingualism works. Although the effects are quite small for young adults (Bialystok et al., 2005), they are larger in older age, contributing to postponement of symptoms of dementia with aging (Bialystok, 2021). Crucially, bilingualism is almost never associated with poorer cognitive outcomes. Put this way, the essence of the controversy is in the reductionism that has led to the expectation that a simplistic definition of bilingualism must lead to improved cognitive test scores, and when it does not, the entire argument is rejected. Masks alone will not end a pandemic. What is needed is a multifactor approach to bilingual experience that takes account of which other slices are in the package and where the holes are on the slice of bilingualism.

## Author Contributions

The author confirms being the sole contributor of this work and has approved it for publication.

## Funding

Preparation of this manuscript was supported by Grant A2559 from the Natural Sciences and Engineering Research Council, Canada.

## Conflict of Interest

The author declares that the research was conducted in the absence of any commercial or financial relationships that could be construed as a potential conflict of interest.

## Publisher's Note

All claims expressed in this article are solely those of the authors and do not necessarily represent those of their affiliated organizations, or those of the publisher, the editors and the reviewers. Any product that may be evaluated in this article, or claim that may be made by its manufacturer, is not guaranteed or endorsed by the publisher.
